# Effect of a Health Coach Intervention for the Management of Individuals With Type 2 Diabetes Mellitus in China: A Pragmatic Cluster Randomized Controlled Trial

**DOI:** 10.3389/fpubh.2018.00252

**Published:** 2018-09-19

**Authors:** Anna Chapman, Colette J. Browning, Joanne C. Enticott, Hui Yang, Shuo Liu, Tuohong Zhang, Shane A. Thomas

**Affiliations:** ^1^School of Nursing and Midwifery and Centre for Quality and Patient Safety Research, Deakin University, Geelong, VIC, Australia; ^2^Monash Health, Clayton, VIC, Australia; ^3^International Institute for Primary Health Care Research, Shenzhen, China; ^4^Research School of Population Health, Australian National University, Acton, ACT, Australia; ^5^School of Primary and Allied Health Care, Monash University, Notting Hill, VIC, Australia; ^6^School of Clinical Sciences, Monash University, Dandenong, VIC, Australia; ^7^Key Laboratory of Carcinogenesis and Translational Research, Beijing Office for Cancer Prevention and Control, Peking University Cancer Hospital and Institute, Beijing, China; ^8^School of Public Health, Peking University Health Science Centre, Beijing, China

**Keywords:** type 2 diabetes, China, health coaching, motivational interviewing, cluster randomized controlled trial, pragmatic trial

## Abstract

**Aim:** To determine the effect of a health coach intervention for the management of glycemic control, as well as physiological, psychological and self-care outcomes of patients with type 2 diabetes mellitus (T2DM), compared with usual care.

**Methods:** This pragmatic cluster RCT was conducted in the Fengtai district of Beijing from August 2011 to December 2013. Forty-one community health stations (CHSs) were cluster randomized (stratified geographically, 1:1 ratio) and eligible, randomly selected T2DM patients were sequentially contacted by CHSs. Control participants received usual care according to the Chinese Guideline for Diabetes Prevention and Management. Intervention participants received 18-months of health coaching based on principles of Motivational Interviewing (MI) plus usual care. Medical and pathology fees were waived for both groups. Outcome assessment was performed at baseline, 6, 12, and 18-months. The primary outcome was glycated hemoglobin (HbA1c); secondary outcomes encompassed a suite of physiological, psychological and self-care measures.

**Results:** No differential treatment effect was found at 18-months for HbA1c (*adj. difference* −*0.07, 95% CI* −*0.53 to 0.39, p* = *0.769*) or any specified secondary outcomes. Interestingly, both groups displayed a statistically and clinically significant within-group improvement of the same magnitude at 18-months for HbA1c (*intervention: mean change* −*3.65, 95% CI* −*3.92 to* −*3.37; control: mean change* −*3.38, 95% CI* −*3.67 to* −*3.08)*.

**Conclusions:** The lack of differential treatment effects observed indicate that it may be premature to recommend the routine delivery of health coach interventions based on MI principles for the management of T2DM in China. However, the large, comparable within-group improvement in mean HbA1c promotes the establishment of free, regular clinical health assessments for individuals with T2DM in China.

Trial Registration: ISRCTN registry - ISRCTN01010526 (https://doi.org/10.1186/ISRCTN01010526)

## Introduction

Diabetes Mellitus (DM) has emerged as a global health concern affecting ≥425 million adults worldwide (1), and an astonishing 25% of these are individuals in China. Recent estimates indicate that 114.4 million adults in China had DM in 2017, and this is projected to reach 119.8 million by 2045 (1). Such large and increasing numbers of DM pose significant challenges to the Chinese health care system, with direct annual costs recently estimated to be Int$170 billion (2). Effective DM management approaches are therefore key goals for China.

Type 2 diabetes mellitus (T2DM), the most prevalent form of DM, “is a complex, chronic condition that requires effective long-term medical management to prevent or postpone chronic complications” (3). To ensure individuals with T2DM receive the wide range of support that is required to manage the physical, psychological and social impacts of their condition, the combination of high-quality clinical care, and self-management support are fundamental (4). However, in China, T2DM management approaches are generally not patient-centered. Alternatively, doctors in China predominantly focus on the pharmaceutical management of T2DM, and generally disregard the facilitation of behavior change to control T2DM-related outcomes (5).

China is currently in the process of major health system reform, and the delivery of health care to individuals with T2DM is in transition. A key focus of these reforms is the establishment of a community-based primary health care system that is affordable, accessible and of high-quality (6). The most recent version of the Chinese Guideline for Diabetes Prevention and Management supports these reform goals by prioritizing the long-term management of T2DM, shifting away from an acute-centered care model that has traditionally been delivered by Chinese hospitals (7). The guideline further recommends the delivery of psychological care to assist individuals in adjusting to a new-diagnosis, and to support adherence to lifestyle changes (7).

A number of T2DM management approaches have been implemented internationally to support individuals in the self-management of their condition. The utilization of peer support programs (8), web-based interventions (9), diabetes self-management education programs (10), and psychological interventions (11) have all been found to have direct benefits for individuals with T2DM. A recent meta-analyses of randomized controlled trials (RCTs) in China similarly supported the use of psychological therapies, namely cognitive behavioral therapy, client-centered therapy, and motivational interviewing (MI) for the improvement of glycemic and psychological outcomes of T2DM (12). However, only three studies were included in the meta-analysis for MI (13–15); validity, as assessed through risk of bias (16), was unclear for the majority of included studies; and only two studies utilizing MI were conducted within the community health setting (14, 17).

Given the lack of methodologically robust studies utilizing MI within the community health sector in China, and the Chinese governments' strong commitment to primary care reform, this study aimed to assess the effectiveness of a T2DM management intervention that utilized health coaches trained in MI within an urban community health setting in China. Based on findings observed in previous meta-analyses (11, 12), it was hypothesized that compared to usual care, the structured health coach intervention would lead to improved physiological, psychological, and self-care T2DM-related outcomes. This paper will report on the final outcome assessment of the trial at 18-months; results of both the pilot study (18) and 12-month pre-results (19) are available elsewhere.

## Methods

### Trial design and participants

A pragmatic, cluster RCT design was implemented from August 2011 to December 2013 within Community Health Stations (CHSs) in the Fengtai district of Beijing, China. This district lies to the southwest of central Beijing and covers an area of approximately 305 km^2^ (20). The utilization of a cluster design was primarily selected to minimize experimental contamination between individuals in the control and intervention groups (health professionals & participants). The study protocol was approved by the Monash University Human Research Ethics Committee (CF11/2657 - 2011001550). All participants gave written informed consent in accordance with the Declaration of Helsinki.

All government-owned and administered CHSs in Fengtai district were identified as eligible clusters, resulting in an overall cluster sample size of 42 CHSs. Prior to randomization, consent for CHS participation was obtained from the Fengtai Health Bureau, the body which governs all eligible CHSs.

To reduce risk of recruitment bias, eligible participants were identified and recruited prior to cluster randomization. A blinded, independent person at each of the 42 CHSs identified eligible participants from medical records. Patients with an established T2DM diagnosis were eligible if they were receiving care at a participating CHS; aged ≥50 years; and lived in Fengtai. Exclusion criteria included a medical condition that precluded adherence to recommendations (e.g., terminal cancer), or the inability to provide informed consent. No additional exclusion criteria were applied due to the pragmatic nature of the trial.

### Sample size

Sample size calculations have previously been reported (19). In brief, sample estimates assumed 42 clusters, a participant attrition rate of 20%, intracluster correlation coefficient of 0.05, power at 80%, alpha of 0.05, and an expected standardized effect of 0.32 for the primary outcome (HbA1c) measured on a continuous scale. This estimated a total of 726 participants were required. Small CHSs aimed to recruit 15 participants, while large stations aimed to recruit 25 participants per station. Consequently, the overall number of participants targeted for recruitment was 780 (control: 395: intervention: 385).

### Stratification and randomization

Eligible participants from each CHS were firstly stratified by gender to achieve a balance of men and women. This was performed as gender-specific health disparities have previously been observed among individuals of Chinese descent with regard to DM management and DM-related outcomes (21). Participants were then randomly sampled by computerized random allocation software, resulting in an ordered list of participants (for each CHS; by gender) to be contacted for consent. Because the socio-demographic profile of individuals residing in Fengtai varies according to locality, CHSs were then stratified by geographic location to achieve a balance of groups. Prior to cluster randomization, one CHS permanently shut down; resulting in a total of 41 CHSs available for randomization. Clusters were randomized into control (*n* = 20) or intervention (*n* = 21) groups using block randomization by computerized random allocation software. This procedure was performed at a central location by an independent person, and all clusters were coded to ensure the block randomization was blinded.

### Participant recruitment

To ensure the required participant sample size was met after the closure of one CHS, one neighboring CHS was required to recruit double the participant numbers (i.e., 30 instead of 15). A recruitment officer at each CHS sequentially contacted and invited individuals to participate in the study. If an individual declined, the reason provided was to be recorded.

All interested participants were provided with a consent form and explanatory statement. Once consent forms were received, participants were allocated an identification number and were instructed to return to their CHS for the baseline clinical health assessment at a specified date and time.

Participants were notified that payment would not provided for participation in the study, but medical fees (consultation and pathology fees) associated with the study would be waived. Despite China having near-universal health insurance coverage, individuals commonly experience out-of-pocket expenses for both pharmaceutical and medical care (22).

### Outcome assessment

Patient level outcomes were assessed at baseline, 6, 12, and 18-months via a clinical health assessment and an interviewer-administered questionnaire. An interviewer-administered mode was chosen in preference to a self-administered mode due to potential literacy problems within the participant group. In 2010, 26.09% of Chinese adults aged >65 were estimated to be illiterate (23).

The primary outcome measure was glycated hemoglobin (HbA1c). Secondary physiological outcomes included weight, body mass index (BMI), systolic and diastolic blood pressure (BP), waist and hip circumference, and fasting blood samples [fasting plasma glucose (FPG), triglycerides, and total, high-density lipoprotein (HDL), and low-density lipoprotein (LDL) cholesterol]. The suite of psychological and self-care secondary outcome measures comprised psychological distress [Kessler 10 (K10)], quality of life (WHOQoL-BREF), diabetes self-care activities (SDSCA), and diabetes management self-efficacy (C-DMSES). All outcomes were assessed by CHS doctors and nurses specifically trained in data collection for the trial, according to the study protocol (24) (except LDL cholesterol, which utilized a direct method). The classification of elevated physiological outcomes was based on specified information within the Chinese diabetes guidelines (7). Participants in both treatment groups were informed of their physiological results following each assessment.

Participants were asked to fast overnight prior to each clinical health assessment for ≥8 h. Due to the pragmatic nature of this trial, data collectors were unblinded to group allocation; however, laboratory technicians were blinded. Analysis of all blood samples was performed at the Fengtai Center for Disease Control and Prevention Laboratory, which has certification from the National Center for Clinical Laboratories of China.

### Intervention

#### Control group

Participants in the control group received usual care only from their CHS. Usual care referred to the standard care as specified in the Chinese Guideline for Diabetes Prevention and Management (7). Although usual care is naturally expected to vary in pragmatic trials between providers and patients, and according to institutional policies, the recommended schedule outlined within the Chinese guideline involves quarterly doctor consultations and biannual physical examinations that include the assessment of HbA1c. The guideline also includes referral to health professionals from multiple disciplines. In order to meet the data collection requirements for this trial, an increase in monitoring was necessary (in addition to that outlined in the guidelines).

#### Intervention group

Participants in the intervention group received a combination of face-to-face and telephone health coaching, plus usual care from their CHS. The health coaching was delivered by experienced clinicians (community nurses, doctors, and psychologists) from each CHS. Before commencing the intervention, health coaches completed a Monash University certified training program in coach-assisted chronic disease management. This entailed a pre-workshop learning phase which included the study of key concepts in patient-centered communications, health psychology, epidemiology of key targeted illnesses and conditions, MI and behavior change, program evaluation, clinical outcome measurement, and the intervention protocol (24). This was followed by a 2-day intensive MI workshop that introduced health coaches to the rationale and framework of MI, and involved the application of core MI skills throughout the behavior change process. This workshop was conducted by a member of the Motivational Interviewing Network of Trainers (25). The workshop was delivered face-to-face in English and an interpreter was used to communicate the content in Mandarin. To ensure health coaches were supported in their MI skill development, refresher workshops were also conducted throughout the 18-month intervention phase. One month after the intervention commenced, health coaches received an additional half-day advanced workshop and were contacted on a quarterly basis for feedback and debriefing.

Health coaches aimed to support participants in achieving the management targets as specified within the Chinese diabetes guidelines (7), with the management of HbA1c < 7% being of primary focus. An intervention manual was used to guide health coaches in utilizing existing local recommendations and guidelines. Variation in intervention delivery was expected due to the pragmatic nature of the trial and the need for health coaches to adapt the manual to local contexts.

**Figure 1:**
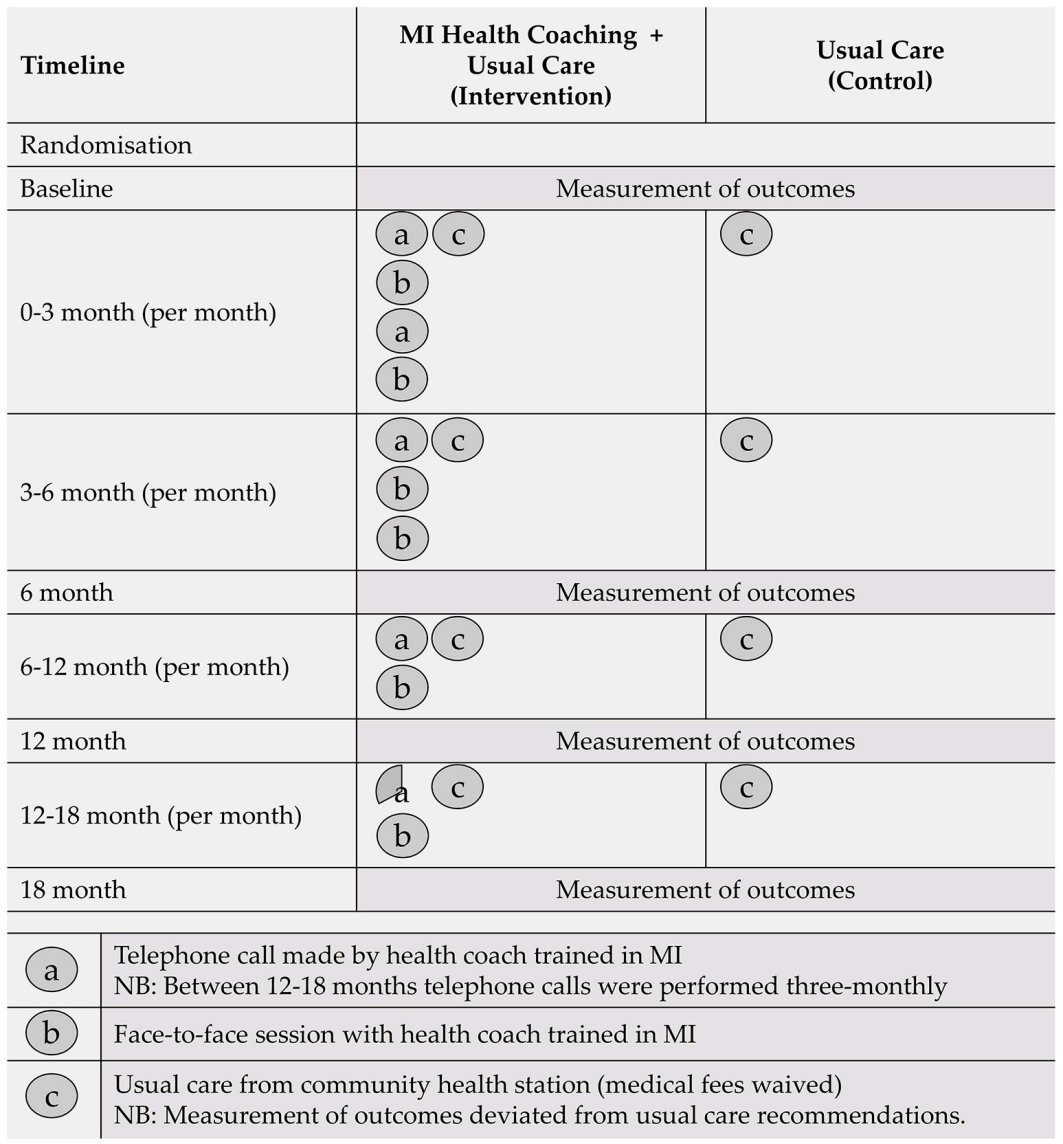
Graphical representation of interventions for each treatment condition.

The first requirement of each health coaching session involved setting the agenda with participants. This was accomplished by asking the participant to determine the most productive place to start the discussion. Once a key issue was identified, health coaches guided the discussion with the fundamental aim of enhancing the participants internal motivation and commitment for change. Initially, participants received two telephone and two face-to-face coaching sessions per month. Session frequency decreased over the 18-month trial period, as depicted in Figure 1.

### Statistical analysis

Statistical analysis was performed using Stata software (v12.0) and a significance level of *p* < 0.05 was used to evaluate statistical significance for both primary and secondary outcomes. Descriptive statistics were used to summarize characteristics (baseline and patterns of mean change over time) of both CHSs and participants. Differences between control and intervention conditions, on all outcomes, were evaluated using multilevel modeling analysis. Specifically, multilevel mixed effects models with robust standard errors were used to assess all continuous outcomes, implemented with the *xtmixed* command. Random effects accounted for within-patient correlation and within-site clustering. Fixed effects included intervention group and time. Primary analysis assessed 6, 12, and 18-month changes in HbA1c. Secondary analyses included all pre-specified continuous outcomes. Model fit was evaluated by comparing AIC values.

Models were adjusted for an *a priori* set of baseline variables, consisting of gender, highest education level, chronic comorbidity category, age group, time since diabetes diagnosis, and residential address socioeconomic status (specific categories provided below Tables 2, 3). Between-group effects were then calculated for all outcomes using the *lincom* command. This procedure estimates an adjusted difference in mean scores between the two treatment groups and its 95% confidence interval. One-way analysis of variance tests were used to estimate ICCs. A missing data analysis was done for each outcome, which consisted of Little's MCAR test to examine patterns of missingness in variables of baseline characteristics and group allocation (Table 1).

**Table 1:** Baseline characteristics of CHSs and participants by study group.

**Baseline characteristics**	**Intervention group**	**Control group**
**Community Health Stations**, ***n*** **(%):**	21 (53.8%)	18 (46.2%)^a^
Population of CHS zone, mean ±*SD*	19,025 ± 10,553	23,884 ± 28,994
Number of annual CHS visits, mean ±*SD* (median)	18,198 ± 16,572 (19,631)	30,862 ± 42,075 (18,730)
Number of CHS doctors, mean ±*SD*	2.9 ± 2.0	3.2 ± 2.3
Number of CHS nurses, mean ±*SD*	3.1 ± 1.8	2.9 ± 1.2
Years since CHS establishment, mean ±*SD*	13.5 ± 6.0	11.4 ± 4.9
**Participants**, ***n*** **(%):**	372 (52.3%)	339 (47.7%)
Age in years, mean ±*SD* (median)	63.7 ± 7.6 (62.2)	64.0 ± 9.0 (63.7)
Female, *n* (%):	191/372 (51.3%)	184/339 (54.3%)
Married (including de facto), *n* (%):	338/371 (91.1%)	299/338 (88.5%)
Retired, *n* (%):	343/370 (92.7%)	309/338 (91.4%)
Secondary/high school education, *n* (%):	266/372 (71.5%)	222/338 (65.7%)
Duration of T2DM in years, mean ±*SD*	10.0 ± 6.5	9.6 ± 6.6
Currently prescribed insulin, *n* (%):	124/371 (33.4%)	99/337 (29.4%)
Co-morbid conditions present, *n* (%):	316/372 (84.9%)	268/339 (79.1%)
Current Smoker, *n* (%):	68/367 (18.5%)	65/321 (20.2%)

*Differences in the participant n denominator for some baseline variables are a consequence of missing data*.

a *Means and SDs for control CHSs based on n = 17. One CHS was an extreme outlier for all variables and subsequently removed for analysis. Baseline characteristics of the excluded CHS are as follows: Population of CHS zone −120,567; Number of annual CHS visits −420,827; Number of CHS doctors −46; Number of CHS nurses −32; Years of CHS establishment −56*.

**Table 2:** Physiological outcomes at baseline and 18 months by study group.

	**Intervention group**			**Control group**		**Available cases analysis**	
**Outcomes**	***N***	**Mean ±*SD***	***N***	**Mean ±*SD***	**ICC^a^**	**Adjusted difference between change scores (95% CI)^b^**	***P*-value**
**PRIMARY OUTCOME**							
**HbA1c (%)**
Target: < 7.0%							
Baseline	359	10.60 ± 2.09	323	10.29 ± 1.71			
18 months	302	6.94 ± 1.65	242	6.99 ± 1.70			
Mean change from baseline to 18 months (95% CI)		−3.65 (−3.92 to −3.37)		−3.38 (−3.67 to −3.08)	0.33	−0.07 (−0.53 to 0.39)	0.769
**SECONDARY OUTCOMES**							
**Weight (kg)**							
Baseline	359	70.13 ± 11.71	333	69.68 ± 10.27			
18 months	283	69.14 ± 11.13	236	67.82 ± 9.77			
Mean change from baseline to 18 months (95% CI)		−0.39 (−0.85 to 0.06)		−0.74 (−1.29 to −0.18)	0.06	0.34 (−0.58 to 1.26)	0.469
**BMI (kg/m**^2^**)**							
Target: < 24 kg/m^2^							
Baseline	359	26.23 ± 3.69	333	26.03 ± 3.42			
18 months	283	25.90 ± 3.42	236	25.64 ± 3.37			
Mean change from baseline to 18 months (95% CI)		−0.15 (−0.32 to 0.02)		−0.27 (−0.48 to −0.05)	0.06	0.12 (−0.23 to 0.46)	0.505
**Waist (cm) Men**							
Target: < 90 cm							
Baseline	176	93.75 ± 9.29	153	92.82 ± 8.49			
18 months	139	93.72 ± 8.85	97	90.73 ± 8.11			
Mean change from baseline to 18 months (95% CI)		+0.11 (−0.75 to 0.98)		−0.89 (−2.07 to 0.29)	0.20	1.31 (−0.57 to 3.20)	0.172
**Waist (cm) Women**							
Target: < 80 cm							
Baseline	186	88.57 ± 9.57	176	90.68 ± 10.02			
18 months	150	88.67 ± 9.37	122	88.38 ± 8.92			
Mean change from baseline to 18 months (95% CI)		+1.01 (−0.02 to 2.04)		−0.90 (−2.28 to 0.48)	0.25	1.38 (−0.74 to 3.49)	0.202
**Hip (cm) Men**							
Baseline	176	102.37 ± 8.69	153	100.23 ± 7.71			
18 months	136	101.62 ± 7.68	101	98.51 ± 6.43			
Mean change from baseline to 18 months (95% CI)		−0.43 (−1.42 to 0.56)		−0.68 (−1.93 to 0.58)	0.23	1.22 (−0.44 to 2.90)	0.150
**Hip (cm) Women**							
Baseline	186	99.95 ± 8.23	179	102.06 ± 8.81			
18 months	155	99.55 ± 7.71	114	98.79 ± 7.99			
Mean change from baseline to 18 months (95% CI)		−0.64 (−1.52 to 0.23)		−1.79 (−3.19 to −0.40)	0.21	1.13 (−0.80 to 3.05)	0.252
**Systolic BP (mmHg)**							
Target: < 140 mmHg							
Baseline	363	129.03 ± 15.14	334	128.46 ± 14.82			
18 months	314	127.50 ± 9.95	262	126.86 ± 11.72			
Mean change from baseline to 18 months (95% CI)		−1.99 (−3.74 to −0.24)		−1.38 (−3.29 to 0.52)	0.07	−0.16 (−2.96 to 2.65)	0.912
**Diastolic BP (mmHg)**							
Target: < 80 mmHg							
Baseline	363	76.91 ± 9.10	334	76.00 ± 8.64			
18 months	315	77.20 ± 6.37	265	74.70 ± 8.06			
Mean change from baseline to 18 months (95% CI)		−0.25 (−1.34 to 0.84)		−0.51 (−1.79 to 0.76)	0.07	1.01 (−1.36 to 3.39)	0.404
**Fasting Plasma Glucose (mmol/L)**							
Target: 4.4–7.0 mmol/L							
Baseline	367	8.27 ± 2.70	330	8.13 ± 2.74			
18 months	314	7.47 ± 2.88	260	7.31 ± 2.69			
Mean change from baseline to 18 months (95% CI)		−0.83 (−1.19 to −0.47)		−0.98 (−1.37 to −0.59)	0.09	0.30 (−0.36 to 0.96)	0.370
**Total Cholesterol (mmol/L)**							
Target: < 4.5 mmol/L							
Baseline	363	5.46 ± 1.12	326	5.40 ± 1.19			
18 months	314	4.86 ± 1.17	259	4.83 ± 1.07			
Mean change from baseline to 18 months (95% CI)		−0.58 (−0.72 to −0.44)		−0.61 (−0.77 to −0.45)	0.02	0.03 (−0.16 to 0.22)	0.748
**Triglycerides (mmol/L)**							
Target: < 1.7 mmol/L							
Baseline	363	1.91 ± 1.53	326	1.82 ± 1.43			
18 months	314	1.59 ± 1.05	259	1.69 ± 1.62			
Mean change from baseline to 18 months (95% CI)		−0.34 (−0.50 to −0.17)		−0.20 (−0.37 to −0.03)	–	−0.06 (−0.23 to 0.11)	0.509
**LDL Cholesterol (mmol/L)**							
Target: < 2.6 mmol/L							
Baseline	367	3.70 ± 1.21	329	3.56 ± 1.13			
18 months	314	3.18 ± 1.05	259	3.07 ± 0.90			
Mean change from baseline to 18 months (95% CI)		−0.53 (−0.67 to −0.39)		−0.55 (−0.70 to −0.40)	0.20	−0.00 (−0.18 to 0.17)	0.979
**HDL Cholesterol (mmol/L) Men**							
Target: >1.0 mmol/L							
Baseline	178	1.04 ± 0.31	151	1.08 ± 0.27			
18 months	146	1.20 ± 0.30	117	1.23 ± 0.27			
Mean change from baseline to 18 months (95% CI)		+0.18 (0.13 to 0.23)		+0.16 (0.10 to 0.22)	0.24	0.01 (−0.06 to 0.08)	0.706
**HDL Cholesterol (mmol/L) Women**							
Target: >1.3 mmol/L							
Baseline	189	1.17 ± 0.35	179	1.17 ± 0.32			
18 months	168	1.25 ± 0.30	142	1.27 ± 0.27			
Mean change from baseline to 18 months (95% CI)		+0.09 (0.04 to 0.14)		+0.11 (0.05 to 0.16)	0.33	0.02 (−0.06 to 0.10)	0.569

*Where relevant, treatment targets for each outcome are provided to assist the interpretation of values. With the exception of HDL cholesterol, lower scores indicate improvement*.

a *ICC, intracluster correlations indicated with a “–” were truncated at zero*.

b *Differences between groups were estimated by multilevel regression, adjusting for clustering and baseline covariates of age group (< 60 years/≥60 years), gender (male/female), chronic comorbidity category (diabetes only/diabetes plus other/s), time since diabetes diagnosis (< 5 years/5–9 years/10–14 years/≥15 years), education level (primary school or less/secondary or high school/tertiary education), and residential address socioeconomic status (Developed areas/Developing areas/Less developed areas)*.

**Table 3:** Psychological and self-care outcomes at baseline and 18 months by study group.

	**Intervention group**			**Control group**		**Available cases analysis**	
**Outcomes**	***N***	**Mean ± SD**	***N***	**Mean ± SD**	**ICC**	**Adjusted difference between change scores (95% CI)^a^**	***P*-value**
**PSYCHOLOGICAL DISTRESS (SCORE RANGE 10–50; RISK CATEGORIES**<**20 NO/LOW; 20–24 MILD; 25–29 MODERATE; 30–50 SEVERE)**							
Baseline	362	15.31 ± 6.85	328	14.97 ± 6.24			
18 months	310	16.98 ± 5.92	263	15.10 ± 5.59			
Mean change from baseline to 18 months (95% CI)		+1.72 (0.80 to 2.63)		+0.30 (−0.66 to 1.26)	0.20	0.56 (−2.19 to 3.31)	0.688
**DIABETES MANAGEMENT SELF-EFFICACY (SCORE RANGE 0–200)**							
Baseline	366	159.32 ± 32.99	334	158.96 ± 34.85			
18 months	311	155.69 ± 31.68	261	158.46 ± 28.05			
Mean change from baseline to 18 months (95% CI)		−4.90 (−9.32 to −0.48)		−2.74 (−7.42 to 1.95)	0.16	8.28 (−7.42 to 23.98)	0.301
**SUMMARY OF DIABETES SELF-CARE ACTIVITIES (SCORE RANGES 0–7; REPRESENTING NUMBER OF DAYS ACTIVITY IS PERFORMED)**							
**General Diet**							
Baseline	368	5.37 ± 1.80	337	5.44 ± 1.85			
18 months	314	5.33 ± 1.37	263	5.50 ± 1.31			
Mean change from baseline to 18 months (95% CI)		−0.07 (−0.30 to 0.16)		−0.08 (−0.35 to 0.18)	0.16	0.21 (−0.31 to 0.73)	0.432
**Specific Diet**							
Baseline	368	4.10 ± 1.51	337	4.37 ± 1.62			
18 months	314	4.10 ± 1.12	263	4.29 ± 1.22			
Mean change from baseline to 18 months (95% CI)		−0.01 (−0.22 to 0.20)		−0.12 (−0.36 to 0.13)	0.11	−0.22 (−0.61 to 0.18)	0.276
**Exercise**							
Baseline	369	5.27 ± 2.07	337	5.01 ± 2.13			
18 months	314	5.11 ± 1.74	263	5.07 ± 1.86			
Mean change from baseline to 18 months (95% CI)		−0.21 (−0.47 to 0.05)		−0.11 (−0.41 to 0.19)	0.08	−0.06 (−0.57 to 0.46)	0.830
**Blood Glucose Monitoring**							
Baseline	366	1.46 ± 1.75	336	1.85 ± 1.83			
18 months	313	2.34 ± 1.90	263	2.56 ± 1.97			
Mean change from baseline to 18 months (95% CI)		+0.78 (0.50 to 1.06)		+0.73 (0.42 to 1.03)	0.09	0.03 (−0.62 to 0.69)	0.920
**Foot Care**							
Baseline	368	4.50 ± 2.79	337	4.46 ± 2.62			
18 months	313	4.80 ± 2.42	263	5.30 ± 2.11			
Mean change from baseline to 18 months (95% CI)		+0.25 (−0.09 to 0.60)		+0.84 (0.46 to 1.22)	0.10	0.19 (−0.78 to 1.15)	0.706
**QUALITY OF LIFE (SCORE RANGE FOR EACH DOMAIN 0–100)**							
**Physical Domain**							
Baseline	368	62.62 ± 12.92	338	63.31 ± 13.99			
18 months	314	59.55 ± 11.30	263	62.65 ± 13.21			
Mean change from baseline to 18 months (95% CI)		−3.09 (−4.75 to −1.43)		−1.56 (−3.46 to 0.34)	0.08	−0.36 (−4.79 to 4.08)	0.875
**Psychological Domain**							
Baseline	368	68.87 ± 14.45	336	67.90 ± 16.18			
18 months	313	61.48 ± 15.71	263	67.16 ± 14.64			
Mean change from baseline to 18 months (95% CI)		−7.69 (−9.94 to −5.44)		−1.88 (−4.31 to 0.55)	0.21	−4.16 (−10.98 to 2.66)	0.232
**Social Relationship Domain**							
Baseline	368	64.18 ± 13.95	338	64.82 ± 14.23			
18 months	313	62.55 ± 13.63	263	65.49 ± 13.24			
Mean change from baseline to 18 months (95% CI)		−1.67 (−3.88 to 0.54)		+0.90 (−1.31 to 3.12)	0.12	−0.52 (−4.96 to 3.92)	0.818
**Environment Domain**							
Baseline	368	65.41 ± 14.53	338	63.72 ± 15.95			
18 months	314	59.80 ± 16.40	263	63.81 ± 14.27			
Mean change from baseline to 18 months (95% CI)		−5.72 (−7.99 to −3.45)		−0.48 (−2.79 to 1.83)	0.18	−3.55 (−9.43 to 2.33)	0.237

*Score ranges for each outcome are provided to assist the interpretation of values. With the exception of psychological distress (K10), higher scores indicate improvement in outcome*.

a *Differences between groups were estimated by multilevel regression, adjusting for clustering and baseline covariates of age group (< 60 years/≥60 years), gender (male/female), chronic comorbidity category (diabetes only/diabetes plus other/s), time since diabetes diagnosis (< 5 years/5–9 years/10–14 years/≥15 years), education level (primary school or less/secondary or high school/tertiary education), and residential address socioeconomic status (Developed areas/Developing areas/Less developed areas)*.

## Results

**Figure 2:**
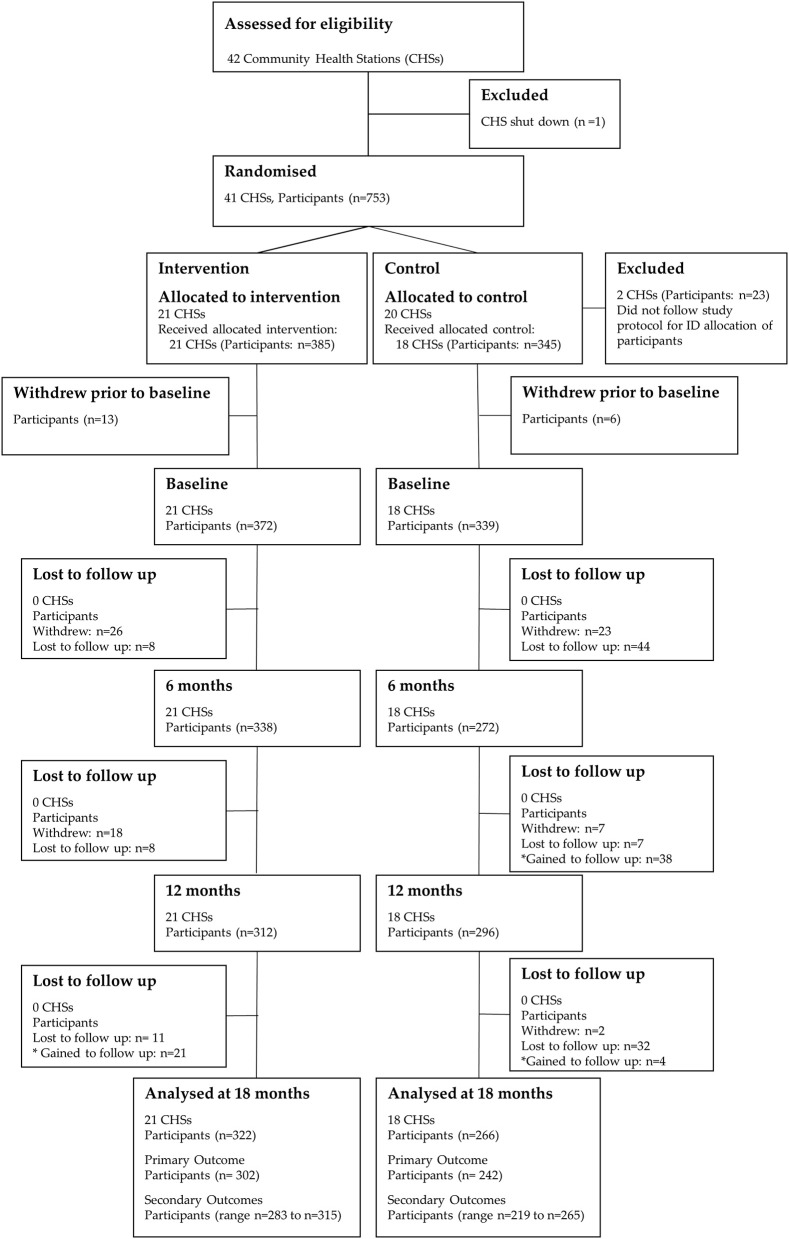
CONSORT flow diagram of community health stations and participants through the 18-month trial. Differences in the participant *n* values for the primary and secondary outcomes at 18-months are due to missing/invalid data.

Between August 22, 2011, and November 15, 2011, 753 participants were recruited into the trial. The trial flow for CHSs and participants is depicted in Figure 2. Of the 20 CHSs randomized to the control condition, two CHSs did not recruit participants according the study protocol. This led to inaccurate ID allocation of participants, and these clusters were subsequently removed from the study prior to baseline data collection (23 participants). Therefore, 39 CHSs [*18 control CHSs (345 participants*) *and 21 intervention CHSs (385 participants)*] were included in the study. No additional CHSs were lost to follow-up throughout the 18-month trial.

Prior to baseline data collection, 19 participants (*intervention: n* = *13; control: n* = *6*) withdrew and did not commence the study. Of the 711 participants (*intervention: n* = *372; control: n* = *339*) who participated in baseline data collection, 588 (*intervention: n* = *322; control: n* = *266*) were included in the analysis at 18-months. The participant attrition from baseline was 17.3% (*intervention: 13.4%; control: 21.5%)*. Analysis of missing data showed that dropouts were missing completely at random (*Little's MCAR test: Chi-Square* = *19.4, df* = *18, p* = *0.4*).

Baseline data were obtained from 39 CHSs (*21 intervention; 18 control*) and 711 participants (*372 intervention; 339 control*) (Table 1). The two trial arms were balanced for all variables at both the cluster and participant level.

With regard to the characteristics of CHSs, the mean population of CHS zone in the intervention and control group was 19,025 ± 10,553 and 23,884 ± 29,994, respectively. The mean number of annual CHS visits was 18,198 ± 16,572 for intervention CHSs and 30,862 ± 42,075 for control CHSs. The doctor to nurse ratio was approximately 1:1 (*intervention: 2.9* ± *2.0:3.1* ± *1.8; control: 3.2* ± *2.3: 2.9* ± *1.2*) and the mean number of years since CHS establishment was >10 years for both groups (*intervention: 13.5* ± *6.0; control: 11.4* ± *4.9*).

At the participant level, mean age for both groups was approximately 64 years (*intervention: 63.7* ± *7.6; control: 64.0* ± *9.0*); and there were slightly more women in the sample (*intervention: 51.3%, 191/372; control: 54.3%, 184/339*) than men. The majority of participants in both groups were married (*intervention: 91.1%, 338/371; control 88.5%, 299/338*), had received secondary school education (*intervention: 71.5%, 266/372; control: 65.7%, 222/338*), and were retired (*intervention: 92.7%, 343/370; control: 91.4%, 309/338*). Mean T2DM duration for both groups was approximately 10 years (*intervention: 10.0* ± *6.5; control: 9.6* ± *6.6*) and roughly one third of participants were prescribed insulin at baseline (*intervention: 33.4%, 124/371; control: 29.4%, 99/337)*. Additionally, the majority of participants in both groups had chronic co-morbid conditions present at baseline (*intervention: 84.9%, 316/372; control: 79.1%, 268/339*) and approximately one-fifth of participants were categorized as current smokers (*intervention: 18.5%, 68/367; control: 20.2%, 65/321*).

Table 2 shows the primary and secondary physiological outcomes; Table 3 the secondary psychological and self-care outcomes. At 18-months, no significant between-group differences were found for the primary outcome measure, HbA1c (*adj. difference* −*0.07, 95% CI* −*0.53 to 0.39, p* = *0.769*), with both intervention and control groups displaying statistically significant improvements (*intervention: mean change* −*3.65, 95% CI* −*3.92 to* −*3.37; control: mean change* −*3.38, 95% CI* −*3.67 to* −*3.08*). Similarly, no differential treatment effects were found at 18-months for any of the secondary physiological, psychological or self-care outcome measures. However, both groups displayed statistically significant within-group improvements at 18-months in FPG (*intervention: mean change* −*0.83, 95% CI* −*1.19 to* −*0.47; control: mean change* −*0.98, 95% CI* −*1.37 to* −*0.59*), triglycerides (*intervention: mean change* −*0.34, 95% CI* −*0.50 to* −*0.17; control: mean change* −*0.20, 95% CI* −*0.37 to* −*0.03*), total cholesterol (*intervention: mean change* −*0.58, 95% CI* −*0.72 to* −*0.44; control: mean change* −*0.61, 95% CI* −*0.77 to* −*0.45*), HDL cholesterol (*men—intervention: mean change* +*0.18, 95% CI 0.13 to 0.23; control: mean change* +*0.16, 95% CI 0.10 to 0.22: women—intervention: mean change* +*0.09, 95% CI 0.04 to 0.14; control: mean change* +*0.11, 95% CI 0.05 to 0.16*), LDL cholesterol (*intervention: mean change* −*0.53, 95% CI* −*0.67 to* −*0.39; control: mean change* −*0.55, 95% CI* −*0.70 to* −*0.40*), and SDSCA-Blood Glucose Monitoring (*intervention: mean change* +*0.78, 95% CI 0.50 to 1.06; control: mean change* +*0.73, 95% CI 0.42 to 1.03*).

Additional within-group significant changes were observed between baseline and 18-months that were not consistent among both treatment groups. With regard to physiological outcomes at 18-months, only intervention participants displayed small yet statistically significant improvements in systolic BP (*mean change* +*0.84, 95% CI 0.46 to 1.22*); while statistically significant improvements in weight (*mean change* −*1.99, 95% CI* −*3.74 to* −*0.24*), BMI (*mean change* −*0.27, 95% CI* −*0.48 to* −*0.05*), and hip circumference for women (*mean change* −*1.79, 95% CI* −*3.19 to* −*0.40*) were only observed within participants in the control group.

Significant within-group deteriorations in the mean between baseline and 18-months were observed among intervention participants only for a considerable number of psychological outcomes, namely psychological distress (*mean change* +*1.72, 95% CI 0.80 to 2.63*), C-DMSES (*mean change* −*4.90, 95% CI* −*9.32 to* −*0.48*), and the WHOQoL-BREF domains of physical (*mean change* −*3.09, 95% CI* −*4.75 to* −*1.43*), psychological (*mean change* −*7.69, 95% CI* −*9.94 to* −*5.44*), and environment (*mean change* −*5.72, 95% CI* −*7.99 to* −*3.45*). In contrast, a statistically significant within-group improvement in the SDSCA-Foot Care subscale at 18-months was observed among participants in the control group only (*mean change* +*0.84, 95% CI 0.46 to 1.22*).

## Discussion

This study is the first pragmatic cluster RCT to examine the effectiveness of a health coach intervention for the management of physiological, psychological and self-care outcomes of T2DM within the community health sector in China, relative to usual care. The findings obtained found no differential treatment effect for HbA1c or for a series of physiological, psychological or self-care secondary outcomes. As such, the study hypothesis was not supported.

Chief among the key findings observed were the significant within-group improvements in mean HbA1c between baseline and 18-months for participants from both treatment groups. In addition to being statistically significant, the comparable change in mean HbA1c from approximately 10% at baseline to within the optimal range of < 7.0% at 18-months, is of clinical significance. Glycemic control is central to T2DM management, and evidence from various landmark studies indicate that lower HbA1c levels are associated with delayed onset or progression of microvascular complications (26). Epidemiological analyses have further revealed a curvilinear relationship between HbA1c and microvascular complications (27). This relationship indicates that the greatest number of complications will be prevented, at a population level, by shifting individuals from very poor glycemic control to fair/good glycemic control. The improvements observed for HbA1c in the present study, if sustained, therefore have the potential for reductions to be noted in microvascular complications.

Although significant within-group changes were noted for numerous secondary outcomes at 18-months, it is unlikely that any of the observed changes will translate into clinical significance. The magnitude of mean change was relatively small for all secondary outcomes; mean values for the majority of statistically significant physiological outcomes remained outside of optimal range at 18-months (except triglycerides and HDL cholesterol for men); and the within-group deteriorations in mean values for psychological and self-care outcomes for participants in the control group (i.e., psychological distress) were not representative of a clinical shift in risk categories. Clinical significance could be argued for the improvements observed in systolic BP among intervention group participants and improvements in triglycerides among participants in both treatment groups. However, the objectivity of triglyceride levels as a clinically relevant outcome is debatable (28); and the clinical significance of systolic BP improvements < 140 mmHg is unclear (29).

The clinically significant improvement in HbA1c among both treatment groups may partially be explained by the regular clinical monitoring and resultant feedback to participants following each clinical health assessment. Most individuals with T2DM in China do not regularly attend specific appointments to manage their condition. The Diabcare-Asia (China) study determined that only 50% of patients with diagnosed DM had a HbA1c test in the preceding 12-months (30), despite Chinese diabetes guidelines prescribing biannual HbA1c assessments (7). Precise health service utilization records were unable to be retrieved for the time-period before the present study, but the numerous physiological outcomes with mean values outside optimal ranges at baseline (in particular HbA1c >10%) indicates that both groups of participants were either not accessing CHSs for T2DM management; were not fully adherent to the T2DM self-care regimen; or were experiencing suboptimal T2DM care prior to participation in this study. Participation in this trial required individuals to undergo health checks at more regular intervals than that outlined in the usual care recommendations. By informing participants of their T2DM health status on a more regular basis, it may have motivated individuals and their healthcare professionals to be more attentive to their health than otherwise expected, thus modifying “usual care.”

Furthermore, to maximize participation, medical fees associated with participation in the present study were waived for both treatment groups, further modifying the “usual care” condition. Out-of-pocket healthcare costs are a well-documented barrier to the accessibility and satisfaction of healthcare services in China (31). Out-of-pocket costs are also higher for older (>60 years), retired individuals who are not eligible for the Urban Employee Basic Medical Insurance scheme (22). As >90% of participants in the present study were retired at baseline, the majority of study participants would usually have been required to incur significant out-of-pocket costs to manage their T2DM. Possible consequences of the adjustments to usual care may have been improvement in adherence to the T2DM self-care regimen; increased CHS attendance; as well as the initiation of medication to manage uncontrolled variables.

A further explanation for the comparable improvement in HbA1c within both treatment groups is the possible contamination between clusters. Although every effort was made to prevent contamination, the trial received considerable media attention during the intervention phase, which might have contributed to participants and health coaches altering their usual behavior. Furthermore, all participating CHSs are administered and managed by the Fengtai Health Bureau. This was an unavoidable circumstance of the study setting but is one that may have affected and blurred the delivery of the intervention and control procedures. Lastly, the “Hawthorne Effect,” typically described as the human tendency to improve performance because of the awareness of being studied (32), may also have contributed to the improvements noted in both treatment groups.

The lack of clinical relevance for the statistically significant findings were primarily due to the small magnitude of changes for each of the secondary outcomes. The small extent of improvements observed in the present trial may be related to the pragmatic nature of the trial. The broad eligibility criteria that was utilized resulted in a diverse population of individuals with T2DM that more closely represents typical clinical practice in China. The resulting heterogeneity of participants could have diluted the observed treatment effects which can occur in pragmatic trials (33). Additionally, the potential variability of intervention delivery between each CHS, and the abovementioned considerations of possible contamination between intervention and control CHSs may have further contributed to a dilution of treatment effect. If this study utilized a traditional RCT design under heightened experimental conditions, observed treatment effects may have potentially been larger.

To date, no cluster RCTs in China have examined the effect of health coaching based on MI principles for the management of individuals with T2DM. A recent systematic review and meta-analyses identified five studies that utilized MI, all of which adopted a traditional RCT design (12). As previously noted, the effects of these studies provide some evidence to support the use of MI for the improvement of glycemic control in patients with T2DM; however studies primarily assessed outcomes at 6 months; validity, as assessed through the risk of bias was unclear for the majority of studies; and baseline HbA1c values were lower (7–8%) than that observed in the present study (>10%). Additionally, only two (14, 17) of the five studies were conducted within a community health setting [one of which assessed HbA1c (14)]. These clinical and methodological variations consequently impede the ability for meaningful comparisons to be drawn between the present study and those previously conducted in China.

Internationally, three cluster RCTs have assessed the effectiveness of MI for the improvement of T2DM-related outcomes (34–36). The between-group findings observed in all three trials are largely consistent with that observed in the present study, with no differential treatment effects observed. Despite the limited number of international cluster RCTs published, several traditional RCTs have been conducted in international settings that have assessed the effectiveness of MI for the management of T2DM-related outcomes. Some of these studies have observed a differential treatment effect favoring the intervention with regard to glycaemic control (37), fat intake (38, 39), physical activity (38), weight (40), waist circumference (38), systolic BP (41), DM self-care activities (42), and DM-related knowledge (43). However, for the majority of outcomes in the majority of studies, differential treatment effects have not been found (36, 37, 43, 44); a result similar to that observed in the present trial.

A key strength of the present study was that it was implemented as a pragmatic trial, specifically tailored to be delivered in real world CHSs in urban China, hence maximizing external validity. The cluster design minimized contamination between CHSs, and stratification and randomization procedures for both participants and CHSs minimized selection bias and increased generalizability of the results to other populations of patients with T2DM in urban China.

A limitation of the present study is that intervention fidelity has not been adequately assessed as yet; hence, we have not been able to distinguish between participants with respect to quality of MI received. In the current trial, it is possible that health coaches had not reached an appropriate standard to be effective MI health coaches, despite increasing their skills. All coaching sessions were audio-recorded throughout the 18-month trial and future research is planned for the analysis of treatment integrity. Until this piece of work is performed, it would be unjustified to conclude that MI is ineffective in the management of T2DM in China.

The pragmatic design also caused data collection to be performed by multiple data collectors from all CHSs. While all data collectors were received extensive training on the study protocol, quality of data varied, resulting in higher levels of missing data than expected. Additionally, outcome assessors were not blinded in the present study. While the lack of blinding in pragmatic trials can lead to reduced internal validity; the external validity is enhanced, and consequently improves the generalizability of findings to clinical settings in which they would be applied (33).

Despite the noted limitations, and the lack of differential treatment effects observed, important implications can be drawn from the findings of the present study. The comparable shift in mean HbA1c among both groups from >10% at baseline to within the optimal range of < 7% at 18-months is a clinically relevant outcome that promotes the establishment of free, regular clinical health assessments for individuals with T2DM in China. The establishment of such a monitoring program would also fit within the current healthcare reform, which aims to address inequitable access to healthcare services. Existing evidence indicates that healthcare insurance can improve the health outcomes of some population subgroups in China, including those of older people (45). Given that older people are associated with the highest burden of T2DM cases in China, and are more likely to be retired with limited finances, this population group would particularly benefit from regular monitoring that is free of cost.

In all countries, we are struggling with the effective management of chronic disease. To date, there is little evidence globally for the long-term success of large-scale interventions applying behavioral management of diabetes. However, we must keep researching this important area in order to alleviate the large burden of disease resulting from sub-optimal diabetes management. This study represents a foundational step toward the implementation of rigorously designed psychological interventions in China, specifically targeting T2DM. Given the combination of China's increasing burden of T2DM and the governments' strong commitment to healthcare reform, the opportunities for meaningful contributions in the field of chronic disease management in China are manifest. The Chinese government and Chinese Medical Association are striving to adopt best practice medical management and the continued examination of the effectiveness of psychological interventions in T2DM management is a worthy and important element.

## Data sharing

All available data is presented within this manuscript.

## Author contributions

CB and ST led the conception of the study. CB, ST, and TZ obtained research and operational funding, and HY participated. AC, CB, and HY led the design of the study protocol. AC drafted the article and all the authors contributed to critical revision. SL, AC, HY, and TZ coordinated the data collection and data cleaning. JE led the statistical analysis of the data and AC contributed. All the authors participated in the interpretation of the trial data. All the authors, internal and external, had full access to the study data, and take responsibility for the integrity of the data and the accuracy of the data analysis. All the authors have approved the final version of the manuscript.

### Conflict of interest statement

AC, CB, ST, and SL are Editors of the research topic: Chronic Illness and Ageing in China; they had no involvement in the review of this manuscript. The remaining authors declare that the research was conducted in the absence of any commercial or financial relationships that could be construed as a potential conflict of interest.
